# Systematic Review: Intervention Strategies for Treating Relational Aggression in Female Juvenile Offenders and At-Risk Female Youth

**DOI:** 10.1016/j.jaacop.2024.04.003

**Published:** 2024-05-15

**Authors:** Jenny Magram, Erica Ackerman, Claire Stafford, Tom D. Kennedy

**Affiliations:** Nova Southeastern University, Fort Lauderdale, Florida

**Keywords:** at-risk female adolescents, intervention, female juvenile offenders, relational aggression, treatment

## Abstract

**Objective:**

Female juvenile offenders commonly display a distinct form of aggression, known as relational aggression, which demands its own dedicated focus and specialized intervention approaches, as exemplified by the limited yet steadily growing body of research on the issue. This review set out to explore current relational aggression interventions specifically designed for incarcerated female youth, which was subsequently broadened to include at-risk female adolescents.

**Method:**

In stage 1, the effectiveness of intervention strategies targeting relationally aggressive behaviors in female juvenile offenders was systematically reviewed. As so few studies existed in the peer-reviewed literature, in stage 2, a review was conducted with a broader scope examining intervention strategies only with at-risk female adolescents.

**Results:**

The electronic databases JBI EBP, PsycINFO, and PubMed/MEDLINE were searched for the systematic review. At stage 1, 16 full-text articles were reviewed for quality, and of these articles, 13 were excluded due to sample population, outcomes, and lack of measuring correlates of relevant behavior. At stage 2, 12 full-text articles were reviewed for quality, and of these articles, 6 were excluded for the above-mentioned reasons.

**Conclusion:**

There are promising advancements in the development and implementation of interventions tailored to attenuate relationally aggressive behaviors in female youth deemed at risk or currently in the juvenile justice system.

## Plain language summary

Female juvenile offenders commonly display a distinct form of aggression, known as relational aggression. The effectiveness of intervention strategies targeting relationally aggressive behaviors in female juvenile offenders and subsequently at-risk female adolescents were systematically reviewed in 2 separate stages. It was found that there were promising advancements in the development and implementation of interventions tailored to attenuate relationally aggressive behaviors observed in young females deemed at-risk or currently in the juvenile justice system.

The exposition and treatment of aggression poses a significant challenge due to the nature of this complex and multifaceted behavior. Despite decades of research, the traditional association of aggression with physicality has overshadowed a distinct and increasingly recognized form—relational aggression (RA) that is disparately exemplified within the female population.^1^ Although RA is gaining increased attention, literature focused on treatment strategies specifically for this population is scarce. Consequently, there is limited research dedicated to identifying successful intervention strategies aimed at reducing RA in at-risk and incarcerated female adolescents.

RA is often defined as behavior in which individuals use their relationships as a method to harm or hurt their peers.^1^^,^^2^ Specifically, relationally aggressive behaviors are acts that encompass nonphysical aggression often in the form of manipulation, peer rejection, social exclusion, spreading rumors, and bullying.^3^ Crick and Grotpeter^1^ further define RA as encompassing both direct behaviors (eg, conditional invitations) and indirect acts (eg, silent treatment). Terms such as indirect aggression and social aggression are often used to convey the idea of RA, with indirect aggression being nonconfrontational and inclusive of covert behaviors beyond RA.^4^ Although RA is a widely used term, there is inconsistency in the terminology and definitions of nonphysical aggression.^4^ Therefore, the operationalization of relationally aggressive behavior may vary among studies.

Gender plays a significant role in shaping the type of aggression youth may exhibit, with male and female youth often displaying varied patterns of aggressive behavior.^5^ Female adolescents more often use indirect or covert aggression, better known as RA, as they tend to prioritize interpersonal relationships and social interactions.^6^ Several studies have confirmed that male adolescents tend to display a higher frequency of overt physical aggression.^1^^,^^7^ However, male adolescents often engage in a combination of both physical and RA.^4^ In fact, there is some evidence that male adolescents exhibit levels of RA comparable to female adolescents.^4^ Yet, it is crucial to acknowledge that such disparities may stem from methodological variations in assessment and the use of approaches susceptible to bias, potentially influencing findings. Moreover, studies employing objective methodologies consistently reveal outcomes in which female adolescents demonstrate significantly higher levels of RA than male adolescents.^4^ This is evidenced by Crick and Grotpeter^1^ confirming the distinction between male and female adolescents in displays of relationally aggressive behavior, with female adolescents being found to show significantly more RA than male adolescents.

Female adolescents not only exhibit this behavior more frequently, but also experience unique and distinct consequences compared with male adolescents involved in such behaviors. Female adolescents who perpetrate RA and those on the receiving end of such aggressive acts are more inclined to exhibit internalizing behaviors, such as depression, loneliness, anxiety, somatic symptoms, as well as a tendency to avoid social interactions.^1^^,^^8^, ^9^, ^10^, ^11^, ^12^, ^13^, ^14^, ^15^, ^16^ Alarmingly, female adolescents who engage in relationally aggressive behaviors are more prone to externalizing behaviors, which encompass delinquency, disruptive and antisocial conduct, substance use, and various other forms of aggression.^11^^,^^14^^,^^16^^,^^17^ Unsurprisingly, RA can result in notable adverse effects and is a contributing factor to female juvenile delinquency.^18^

There has been a steady increase in female adolescents within the juvenile justice system.^18^ In fact, female youth constitute a significant percentage of the total juvenile arrests,^19^ with a noteworthy increase occurring over the past 2 decades, rising from 25% in 2000 to 31% by 2019.^20^ As a result of the increased proportion of juvenile cases involving female adolescents, there is a growing interest and investment in gender-responsive services among researchers.^21^, ^22^, ^23^ The need for intervention strategies targeting RA seen in female juvenile offenders is significant, as there is the potential for long-term deleterious consequences. Such consequences can include loss of friendships, loss of support, and damage to familial relationships, which may contribute to future criminal transgressions and the development or exacerbation of mental health issues.^24^ Therefore, it has become increasingly important to examine the current intervention strategies targeting gender-specific aggressive behaviors that are often a source of criminal misconduct.

Despite the need for gender-specific interventions targeting RA in female youth, there is a lack of evidence-based treatment options.^18^ Many of the existing aggression treatments, originally designed for male adolescents, may not adequately address the unique needs of female adolescents. Recent research has illuminated distinct variables associated with aggression in male and female adolescents underscoring the necessity for tailored approaches.^6^^,^^25^, ^26^, ^27^ Hence, it is imperative to develop treatment and psychoeducational groups for female adolescents taking these distinctions into consideration. Although the existing research on this subject matter is sparse, what is available warrants a thorough review to begin building consensus regarding what intervention strategies are both accessible and most effective in treating RA. Therefore, this review explored interventions specifically designed for at-risk and incarcerated female youth with a focus on the effectiveness of said interventions. By doing so, we aimed to establish a foundation of knowledge that will serve as a guide for both future researchers and clinicians, helping to advance the understanding and implementation of effective treatment interventions for female juvenile offenders and at-risk female adolescents.

## Method

A scoping search was conducted to identify any existing reviews or meta-analyses on the topic of RA interventions for female youth. The scoping search identified no existing reviews in this area. Next, a specific protocol was developed and registered with PROSPERO (CRD42023446508) to avoid duplication and ambiguity. Following registration with PROSPERO, JBI EBP, PsycINFO, and PubMed/MEDLINE electronic databases were searched for both stage 1 and stage 2. The Preferred Reporting Items for Systematic Reviews and Meta-analyses (PRISMA)^28^ guidelines were followed to conduct our review. Search terms and operators for the stage 1 database searches included a combination of the following: relational aggression and female juvenile offenders, aggress∗, non-physical bullying or social bullying, social aggression or social exclusion, female, females or girls, minor or youth, justice involved females or female juvenile delinquents, criminal, intervention or treatment, and training. Due to the limited number of articles yielded from the initial search, stage 2 was conducted to broaden the scope of potential articles. This was achieved by adjusting specific search terms to enhance comprehensiveness. Search terms and operators for stage 2 comprised the same key terms as stage 1 except that term female juvenile offenders was replaced with at-risk female adolescents.

References yielded from the initial and subsequent searches were eliminated if they were duplicates, irrelevant by title or abstract, or inaccessible. The remaining references were removed upon further in-depth quality evaluation of the full-text articles. A modified checklist used to construct the inclusion and exclusion criteria for individual studies was adapted from a checklist employed in the systematic review by Forman-Dolan *et al.*,^29^ which was formulated from the comparator and outcome questions of the PICO model.^30^

### Inclusion and Exclusion Criteria

The inclusion criteria were the same for both stages 1 and 2 and were as follows:

1. The study included an RA or relevant psychological behavior determination.
2. Correlates of RA or aggression in general were measured.
3. A sound methodological framework was present with results of the study being directly linked to the aim of the study.
4. Intervention strategies used for treating RA were described.

Exclusion criteria slightly differed by stage in terms of sample population, with stage 1 excluding studies if the observed population consisted of female adolescents who were not juvenile offenders, had not been involved in the criminal justice system, or were older than 18 years of age. The exclusion criterion of age older than 18 years for stage 1 was chosen because this is in line with how the criminal justice system views the distinction between a juvenile and an adult.^31^ Stage 2 excluded studies if the observed population consisted of female participants who were not in the adolescent age range (ages 10-19).^32^ Due to widening the scope and changing our target population of female juvenile offenders to at-risk female adolescents, we modified the sample population exclusion criteria to be compatible with how the general population views the adolescent age range rather than the criminal justice system. Therefore, in stage 2 in which the target population was at-risk female adolescents, the exclusion criteria encompassed a broader age range. The checklist used for appraising the quality of articles for both stages 1 and 2 contains all the exclusion criteria (Table 1).

**Table 1:** Quality Appraisal Checklist for Stages 1 and 2

Inclusion criteria	
**Yes**	
☐	Is the study relevant to the research questions?
☐	**Behavior** (one of the following must be checked “yes”)
	☐ Aggression (shows symptoms as determined by a valid psychometric measurement and/or through observation)
	☐ Relational aggression (shows symptoms as determined through observation and/or information gathered from interview)
	☐ Nonphysical bullying (shows symptoms as determined through observation and/or information gathered from interview)
	☐ Social bullying (shows symptoms as determined through observation and/or information gathered from interview)
	☐ Relational bullying (shows symptoms as determined through observation and/or information gathered from interview)
☐	**Correlation** (must be checked “yes”)
	☐ Must measure correlates of relational aggression (aggression in general)
☐	**Inferential statistics** (both must be checked “yes”)
	☐ Includes a sound methodological framework (ie, a body of methods, a set of procedures, and a discussion of results)
	☐ Were the results directly linked to the aim of the study
☐	**Outcomes** (must be checked “yes”)
	☐ Description of intervention strategies used for treating relational aggression
**Exclusion criteria**	
**Yes**	
☐	**Sample population—stage 1** (any of the following are grounds for exclusion)
	☐ A group that does not consist of female juvenile offenders
	☐ A group of female youth who have not been involved in the criminal justice system
	☐ A group of women 19 years old or older
☐	**Sample population—stage 2** (any of the following are grounds for exclusion)
	☐ A group that does not consist of female adolescents
	☐ A group of female youth who are not involved in the justice system or considered at risk of delinquent behavior
	☐ A group of women 19 years old or older
	☐ A group of girls 9 years old or younger
☐	**No outcomes** (the following is grounds for exclusion)
	☐ No outcomes relating to the sample population
☐	**Type of article** (any of the following are grounds for exclusion)
	☐ Non–peer-reviewed article
	☐ Book review
	☐ Editorial
	☐ Master’s thesis
☐	**Assessing risk of bias** (any of the following are grounds for exclusion)
	☐ Selection bias
	☐ Performance bias
	☐ Detection bias
	☐ Attrition bias
	☐ Reporting bias

## Results

**Figure 1:**
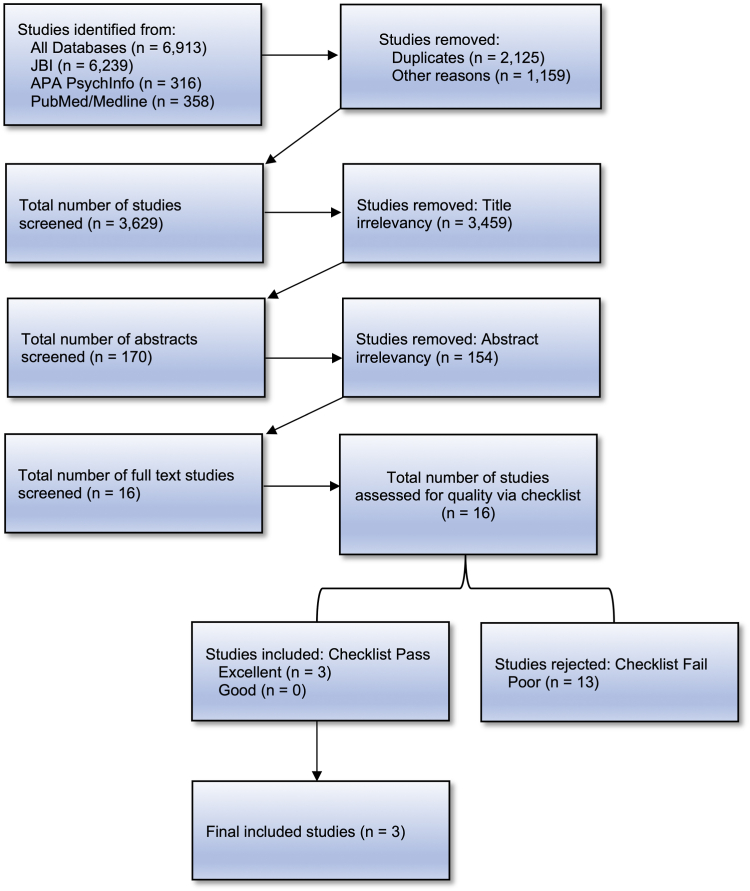
Systematic Review Flowchart for Stage 1

**Figure 2:**
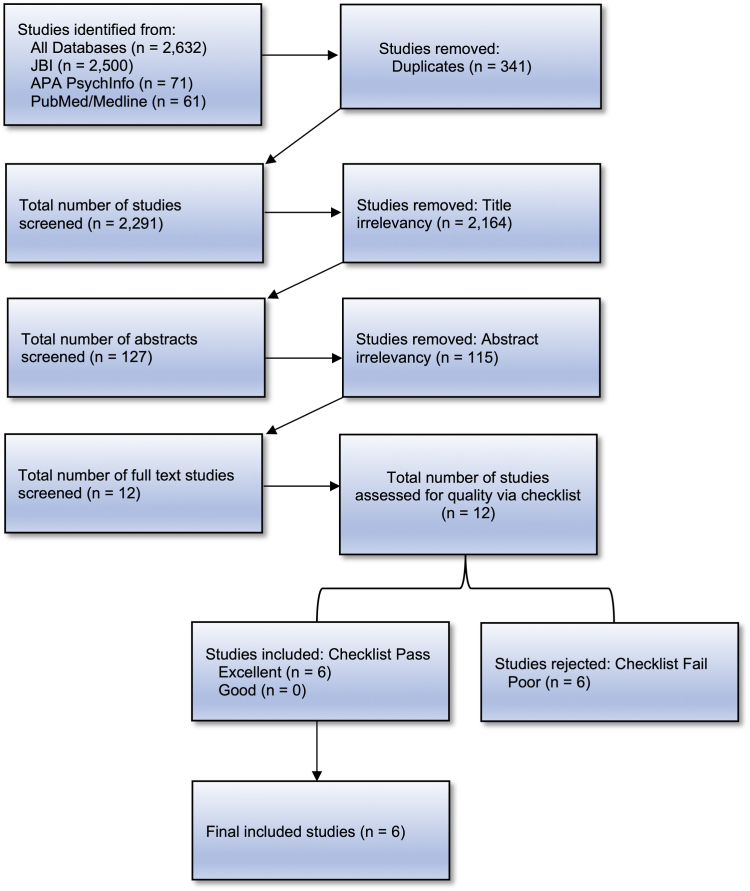
Systematic Review Flowchart for Stage 2

The electronic databases JBI EBP, PsycINFO, and PubMed/MEDLINE were used for the systematic review. Stage 1 generated 3,629 articles, and Stage 2 generated 2,291 articles, excluding duplicates. Two raters independently screened all titles and abstracts for both stages and rated them based on the adapted checklist. Of 16 full-text articles reviewed for quality at stage 1, 13 were excluded due to sample population, outcomes, and lack of measuring correlates of relevant behavior. Of 12 full-text articles reviewed for quality at stage 2, 6 were excluded for the same reasons as stated above. Figures 1 and 2 outline the process for inclusion and exclusion for stages 1 and 2, respectively.

### Methodological Quality

As previously noted, in stage 1, we reviewed the effectiveness of intervention strategies targeting relationally aggressive behaviors in female juvenile offenders. Due to the limited number of articles yielded, a stage 2 review was conducted with a broader scope, examining intervention strategies only with at-risk female adolescents. Therefore, the methodological quality of articles in stages 1 and 2 was assessed separately; 16 studies for stage 1 and 12 studies for stage 2 were assessed for methodological quality by 2 reviewers. The reviewers rated each article depending on whether inclusion or exclusion criteria were satisfied as follows: 0 (poor quality), 1 (good quality), or 2 (excellent quality). All the eligibility criteria were met by 3 studies in stage 1 and 6 studies for Stage 2 and received a rating of excellent. For both stages 1 and 2, zero studies received a rating of good. A rating of poor due to failing to satisfy 4 or more assessment criteria was assigned to 13 studies in stage 1 and 6 studies in stage 2. Therefore, guided by the methodological quality criteria outlined in Table 2, all 3 studies were included in stage 1. In stage 2, 6 studies were integrated.

**Table 2:** Quality Assessment Appraisal for Stages 1 and 2

Quality of study	Studies evaluated	Relevance	Sample population	Measures and outcomes	Inferential statistics
**Stage 1**					
Excellent	Goldstein *et al.*, 2007^24^	Used intervention approach pertinent to RA	Samples comprised female juvenile offenders placed in residential correctional facilities	Various forms of aggression were measured using clearly defined, valid, and reliable instruments	Outcomes describe effects of intervention on RA and/or relevant behavior
	Goldstein *et al.*, 2013^33^				
	Goldstein *et al.*, 2018^35^				
**Stage 2**					
Excellent	Hoffman *et al.*, 2004^34^	Used interventions designed to specifically address RA	Samples comprised female juvenile offenders, at-risk female students, and at-risk/high-risk female adolescents	Various forms of aggression and psychologically related behaviors were measured using clearly defined, valid, and reliable instruments	Outcomes describe effects of intervention on RA and/or relevant behavior
	James *et al.*, 2011^35^				
	Scott, 2012^36^				
	Sieving *et al.*, 2014^37^				
	Splett *et al.*, 2015^38^				
	Stoll-Juredine, 2015^39^				

Note: RA = relational aggression.

### Characteristics of Included Studies

All the studies reviewed for stage 1 were conducted within the United States. The samples consisted of female juvenile offenders who were housed in various residential juvenile justice facilities. One study gathered participants from 3 different residential juvenile justice facilities, while the remaining 2 studies recruited participants from the same post-adjudication juvenile justice facility.

The articles reviewed for stage 2 varied in location from Canada, Ireland, and the United States. The 6 included studies also varied in their design with samples consisting of aggressive and/or high-risk adolescent females, adolescent females exhibiting RA, and at-risk female students. More than half of the samples from the 6 studies were based in an alternative and/or typical educational setting, while others were in a residential facility or a community-based clinic. The type of intervention used, the specific form of aggression addressed and measured, and the outcomes identified in each article for both stages are outlined in Table 3.

**Table 3:** Characteristics of Included Studies for Stages 1 and 2

Reference	Sample	Race/ethnicity	Aggression type	Measures/instrument	Outcomes relevant to RA
**Stage 1**					
Goldstein *et al.*, 2007^24^	Participants (N = 12) were female juvenile delinquents ages 14-18 y	African American (3), American Indian (1), Asian American (1), Latino (2), White (5)	Physical aggression, verbal aggression, and indirect aggression	Aggression Questionnaire, Peer Nomination Measure for Relational and Physical Aggression, NIMH Diagnostic Interview Schedule for Children, and Outcome Expectation Questionnaire	Failed to achieve significant results, but large effect sizes were found
Goldstein *et al.*, 2013^33^	Pilot study: Participants (N = 12) were female juvenile delinquents ages 14-18	N/A	Physical aggression, verbal aggression, RA, and overt aggression	Pilot study: Aggression Questionnaire and NIMH Diagnostic Interview Schedule for Children	Pilot study: Reported a significant result (discrepancy with 2008 findings) with large effect sizes
	Initial trial: Participants (N = 2) were female juvenile delinquents both age 16 y, but only 1 girl was available for posttesting			Initial trial: standardized tests, interviews, self-report measures, peer nomination instruments, and chart reviews	Initial trial: did not report on empirical findings due to small sample size
Goldstein *et al.*, 2018^40^	Participants (N = 57) were female juvenile delinquents ages 14-19 y at time of consent	Asian (2.9 %), Black or African American (69.2%), Hispanic (25.7%), more than one race (22.9%), not Hispanic (74.3%), White (11.4%)	Physical aggression, indirect aggression, RA, reactive aggression, and proactive aggression	Novaco Anger Scale and Provocation Inventory, Aggression Questionnaire, Peer Conflict Scale, Cognitive Emotion Regulation Questionnaire, Intent Attributions and Feelings of Distress Measure, and Outcome Expectation Questionnaire	Yielded significant results
**Stage 2**					
Hoffman *et al.*, 2004^34^	Participants (N = 12) were aggressive adolescent girls ages 12-16 y	Indigenous peoples (2), White (10)	RA, direct aggression, physical aggression, and verbal aggression	Beliefs and Attitudes Scale and Relational and Direct Aggression Scale	Failed to achieve a significant result
James *et al.*, 2011^35^	Participants (N = 92) were at-risk female students ages 16-17 y	N/A	RA and relational bullying	Nonstandardized questionnaire and open-ended questions	Only percentages reported; no statistical analyses conducted
Scott, 2012^36^	Participants (N = 23) were at-risk female students, ages unknown due to lack of reporting from preexisting database	African American (39.1%), American Indian (4.3%), biracial (8.7%), Latino (4.3%), multiracial (4.3%), unknown (group C) (39.1%)	RA and social aggression	Young Adult Social Behavioral Scale	Failed to achieve significant results
Sieving *et al.*, 2014^37^	Participants (N = 239) were high-risk female adolescents ages 13-17 y	Intervention group: American Indian (3%), Asian/Asian American/Asian Pacific Islander (10%), Black/African/African American (45%), Hispanic/Latina (17%), mixed/multiple (19%), White/European American (6%)	RA and physical violence	Adapted from Multisite Violence Prevention Project	Yielded a significant result; small effect size found
		Control group: American Indian (2%), Asian/Asian American/Asian Pacific Islander (13%), Black/African/African American (38%), Hispanic/Latina (8%), mixed/multiple (23%), White/European American (16%)			
Splett *et al.*, 2015^38^	Participants (N = 28) were at-risk female students with a mean age of 13 y	African American (15.2 %), missing data (18.2%), multiracial (9 %), White/non-Hispanic (57.5 %)	RA	Children’s Social Behavior Scale–Self-Report and Children’s Social Behavior Scale–Teacher Report	Yielded a significant result
Stoll-Juredine, 2015^39^	Participants (N = 17) were at-risk female students ages 10-11 y	African American (76.5%), White (23.5%)	RA	Children’s Social Behavior Scale–Self-Report, Children’s Social Behavior Scale–Teacher Report, and Peer Beliefs Inventory	Failed to achieve significant results

Note: N/A = not available; NIMH = National Institute of Mental Health; RA = relational aggression.

### Stage 1

#### Review of Interventions

Although an abundance of literature discusses the topic of RA, there is a paucity of efficacy or effectiveness-based studies designed to reduce relationally aggressive behaviors. The 3 articles reviewed focused on anger management and aggression reduction interventions. It is important to note that these 3 studies were closely related as they were conducted by the same primary author, with 2 of the studies reporting on findings from the same pilot study. These studies specifically implemented Anger Management for Female Juvenile Offenders (AMFJO), which subsequently underwent modifications and revisions as research on the topic advanced and became Juvenile Justice Anger Management (JJAM) Treatment for Girls.

A pilot study conducted in 2007 was the first step in developing an anger management intervention designed for female juvenile offenders, known as AMFJO.^24^ The authors used the Anger Coping program by Lochman *et al.*^41^ as a framework for AMFJO, although changes were made so that it was appropriate to use with female delinquents. The changes involved specific themes such as developmental and population appropriateness, cultural and gender sensitivity, and skills training to reduce RA.^24^

The JJAM intervention was developed to meet the unique needs of female adolescents residing in residential juvenile justice facilities. The authors noted how successful anger management programs are typically based on social information processing (SIP) models,^42^ and thus the JJAM treatment used SIP models as a theoretical basis. Based on SIP models, aggressive children are found to have SIP deficits in encoding, attributions, solution generation, and decision making.^40^, ^42^, ^43^ Furthermore, such children have a tendency to have a heightened arousal in response to anger-producing stimuli, and they may focus on perceived threats and react with physical aggression or RA and hope that their behavior will result in a positive outcome.^40^^,^^43^

The JJAM treatment was based on a modification of an empirically supported treatment known as Coping Power Program (CPP), and it retained key features from the CPP, such as emotional regulation, cognitive restructuring of hostile attributions, and social problem solving, which were viewed as the mechanisms of action for the JJAM study.^40^ It is important to note that JJAM was developed using Participatory Action Research methodology as it allowed for active input throughout the treatment development process from individuals who will engage in or use the manual in the future.^30^^,^^40^^,^^44^ Characteristics of the interventions for stage 1 are outlined in Table 4.

**Table 4:** Characteristics of Interventions for Stage 1

Intervention	Setting	Session/time length	Components
Anger Management for Female Juvenile Offenders (AMFJO)^24^	Residential, female, postadjudication, juvenile justice facility	Two anger management group sessions per week for 9 wk, at 1.5 h per session	Normalizing feelings associated with anger, learning about physiological symptoms of escalating anger, identifying methods for calming down, and generating alternative responses to anger-producing situations
Juvenile Justice Anger Management (JJAM) Treatment for Girls^40^	Residential juvenile justice facility	Two 90-min sessions per week for 8 wk, with groups consisting of 4-6 participants	Psychoeducation on anger, RA and physical aggression, cognitive restructuring to aid in reevaluating assumptions of hostility, identifying cues and triggers of anger, skill-building sessions for managing arousal and preventing aggressive behavior, problem-solving and communication skills, generalizing skills for the future, and point system to promote positive behavior

Note: RA = relational aggression.

#### Effectiveness of Interventions

The primary goal of this stage 1 review was to evaluate the efficacy of interventions regarding the reduction of relationally aggressive behaviors commonly seen in female juvenile offenders. It is important to note that findings from the interventions go beyond the construct of RA, but for the purpose of this review, only results relevant to RA will be discussed. Additionally, it is imperative that the assessment tools used for RA are explained, as it was previously noted that there is inconsistency in the terminology and definitions of nonphysical aggression, and therefore the operationalization of relationally aggressive behavior may vary among studies.^4^ The variation in the conceptualization of RA can lead to the use of a range of measurement techniques to assess the construct, which affects the determination of the effectiveness of interventions reviewed.

For the study using the AMFJO intervention, authors measured RA under the construct of indirect aggression using the Aggression Questionnaire (AQ),^45^ as it was noted that this form of aggression was the best available approximation of RA at the time. Similarly, the authors deploying the JJAM intervention measured RA under the construct of indirect aggression using the AQ. In the JJAM study, statistically significant results were observed when analyzing the AQ indirect aggression subscale, yielding a large effect size (90% CI [0.05, 0.33]).^40^ While results did not reach statistical significance, female participants in the AMFJO treatment condition exhibited improvement in indirect aggression measures after completing the intervention, yielding a large effect size.^24^ The lack of statistically significant results found in relation to indirect aggression scores in the AMFJO study may be due to lack of appropriate power.

The authors of the JJAM study used the Peer Conflict Scale (PCS)^46^ as a secondary measure to assess different forms of relationally aggressive behavior in justice-involved youth. The PCS comprises 6 subscales that include total RA, reactive RA, and proactive RA scales, with findings to suggest that each subscale has good internal consistency.^40^^,^^46^ For the reactive RA subscale on the PCS, female participants in the JJAM treatment condition had significantly lower scores from pretest to posttest, yielding a medium to large effect size (90% CI [0.01, 0.26]). No main effects were found for proactive RA, and a small effect size was found (90% CI [0.00, 0.14]).^40^ Similarly, no main effects were found for the total RA subscale, yet a medium effect size was reported (90% CI [0.00, 0.21]). The measures used in the JJAM study appear to be more comprehensive in quantifying RA than those used in the AMFJO study as the measures used in JJAM assessed subcategories of RA and were able to yield significant results.

The authors of the JJAM study evaluated the mechanisms of action using the Cognitive Emotion Regulation Questionnaire (CERQ)^47^ for emotion regulation, the Intent Attributions and Feelings of Distress Measure (IAFD)^48^ for hostile attribution, and the Outcome Expectation Questionnaire (OEQ)^49^ for social problem solving. The sole emotion regulation subscale score that served as a mediator for the impact of the condition on anger and, consequently, on RA (AQ Indirect Aggression) was the CERQ Catastrophizing T-score.^40^ Measures of social problem solving and anger did not play a significant mediating role in the effect of condition on physical aggression or RA. The pathways from hostile attribution to anger, as potential mediators of the effect of condition on physical aggression and RA, did not show significance. However, a simpler path from the condition to both physical aggression and RA via hostile attribution was noteworthy. Therefore, only changes in hostile attribution bias were supported as a mechanism of action.^40^

### Stage 2

#### Review of Interventions

The objective of stage 2 was to assess the efficacy of diverse interventions aimed at addressing and treating RA in at-risk female adolescents. Authors of the 6 articles reviewed defined the term at risk in a multitude of ways, but for the purpose of this review, we refer to at-risk female adolescents as those who are at an increased risk of developing or maintaining negative behaviors,^50^ such as RA, due to their environment or specific psychological characteristics.^51^ The following interventions were reviewed in terms of their effectiveness for treating at-risk female adolescents: Goodwill Girls (GWG), Growing Interpersonal Relationships through Learning and Systemic Supports (GIRLSS), A Friend In Deed, Prime Time, and A Girl’s Relationship Group (GRG). It should be noted that 2 articles review the same intervention, GWG, yet the intervention was evaluated by 2 separate groups of authors who used different samples, and therefore the articles were evaluated separately.

Given that RA often manifests among at-risk youth within school environments, certain interventions discussed in this section were specifically crafted for integration within educational systems. GWG was developed for students who are at risk of engaging in relationally aggressive behaviors.^36^ The intervention is grounded in psychological theory and solution-focused approaches, which are beneficial for addressing students’ problematic behaviors for which they have been previously reprimanded.^52^

GIRLSS is an empirically informed group counseling intervention designed to treat middle school–age girls who engage in relationally aggressive behaviors.^38^ The GIRLSS intervention was developed based on the Relational Aggression in Girls^53^ curriculum and underwent modifications based on feedback from both participants and group leaders. This led to the construction of the GIRLSS manualized intervention curriculum. The topics covered during group sessions were designed to fixate on the steps of SIP, which proposes that an individual’s ability to process an arrangement of social cues can influence their behavioral response.^38^

The authors noted the importance of targeting deficits in the SIP sequence of aggression in children, which includes attribution retraining. Furthermore, research has shown how group-based interventions are effective in reducing the presence of RA (ie, Making Choices Program,^54^ Friend 2 Friend Program^55^), as is using a multisystemic approach that includes the caregivers in treatment if available. These findings provided the framework for the GIRLSS intervention, as it was noted that the mechanisms of change included attribution styles, normative beliefs about RA, parenting practices and styles, and knowledge of RA and the GIRLSS intervention in general.^38^

The intervention A Friend In Deed educated participants about relationally aggressive behavior, rather than solely focusing on treatment.^35^ Despite this distinction, we, as authors, deemed it crucial to incorporate A Friend In Deed due to its potential to provide valuable insights for shaping future treatments. This intervention was created based on the understanding that change cannot occur without awareness and a true understanding of the issue. Furthermore, the developers of the program acknowledged that adolescents do not always recognize when RA is occurring, and even if they do, they are reluctant to disclose the information to adults.^35^ Following these considerations, A Friend In Deed was designed to educate adolescents about the reasons behind RA, with the goal of helping them to truly comprehend this behavior. James *et al.*^35^ conducted this study with a particular sample as the school administration requested that a specific subset of students receive these education-based lessons due to observed relationally aggressive behaviors.

The 2 remaining interventions were developed for integration within a nonacademic setting. The Prime Time intervention was created with the objective of reducing a variety of at-risk behaviors seen in female adolescents who are at high risk for pregnancy. Sieving *et al.*^37^ conducted a study to assess the effectiveness of interventions in reducing aggression and related behaviors. The authors highlight how adolescents exhibiting aggressive behaviors, particularly RA, are susceptible to various adverse health consequences beyond the risk of unwanted pregnancy. These encompass the emergence of psychological disorders, substance abuse, behavioral issues, and compromised peer relationships.^37^ The design of the Prime Time intervention was guided by the resilience paradigm and social cognitive theory. Additionally, it was created to promote change in psychosocial and behavioral attributes that are commonly associated with involvement in violence.^37^, ^56^, ^57^, ^58^

The second intervention, based in a nonacademic setting, also placed a high importance on the need for positive relationships to reduce relationally aggressive behaviors. GRG^34^ is a group treatment that was developed with a specific focus on RA and used the self-in-relation theory^59^ as well as a feminist understanding of abuse and gender-specific socialization as a theoretical basis.^25^^,^^60^, ^61^, ^62^ GRG aims to provide comprehensive education for female youth, covering their interactions with same-gender peers, verbal aggression and RA, repercussions of violence in their lives, effective strategies for managing challenges, and the cultivation of a positive self-perception.^60^ Characteristics of the interventions for stage 2 are outlined in Table 5.

**Table 5:** Characteristics of Interventions for Stage 2

Intervention	Setting	Session/time length	Components
Goodwill Girls (GWG)^36^^,^^39^	School-based intervention	10 sessions, each lasting approximately 40 min. Spans about 10 wk, with the possibility of completion before or after designated time frame	Social skills development, perspective taking, an individual’s approach to dealing with conflict, and RA
Growing Interpersonal Relationships through Learning and Systemic Supports (GIRLSS)^38^	School-based intervention	About 10 wk, attending a weekly group session lasting 70 min	Factors such as schemas, hostile attribution bias, attachment, and modeling, with caregiver component consisting of workshops and phone consultations. Use of interactive discussions, examples, role-plays, journaling, and goal setting
A Friend In Deed^35^	School-based intervention	Lessons during a double class period, which spans 80 min	Interconnected topics that contribute to emergence of bullying behaviors. Encompassed areas include friendships, jealousy management, conflict resolution, communication skills, power dynamics, popularity, and RA
Prime Time^37^	Clinic-based intervention	Over a period of 18 mo	School and community involvement, emotional and social skills, developing positive relationships with a conscious focus on supportive relationships with individuals in one’s immediate context
A Girl’s Relationship Group^34^	Residential correctional facility	Twice a week for 1 h over a span of 4 wk	Effects of gender-role acculturation on intergender interactions, education on the various forms of aggression, primarily verbal aggression and RA; examining how violence can impact lives in and out of the home, education on coping strategies that are nonaggressive and can be used in stress-inducing interpersonal conflicts, encouraging the development of a positive self-image

Note: RA = relational aggression.

#### Effectiveness of Interventions

As previously mentioned, findings from the interventions go beyond the construct of RA, but for the purpose of this review, only results relevant to RA will be discussed. Additionally, as noted previously, the assessment tools used for RA warrant discussion as there is variation in the conceptualization of RA across studies, which can result in a range of measurement techniques used to assess the construct. This could affect the determination of the effectiveness of interventions reviewed, and as such measures used to assess RA are explained.

The first study using GWG by Scott^36^ examined if the intervention reduced RA and social aggression in female participants who engaged in bullying and other aggressive behaviors. In this study, the Young Adult Social Behavioral Scale (YASB)^63^ was used to assess RA as it was originally designed to measure both healthy and maladaptive behaviors that are seen in friendships and relationships. Interestingly, a second study that used the GWG program by Stoll-Juredine^39^ had similar research objectives yet took a different approach to measuring RA. In this study, the Children’s Social Behavior Scale (CSBS) was used in self-report (CSBS-S) and teacher report (CSBS-T) forms so that both participants and objective observers could report on the frequency of relationally aggressive behaviors.^1^^,^^8^ The participants in this study were assessed using a secondary measure, the Peer Beliefs Inventory (PBI), to evaluate prosocial and antisocial characteristics.^64^

A series of analyses were conducted by Scott^36^ using archival data. Scott^36^ compared the pretest and posttest scores of participants in the treatment conditions using a paired samples t test. A reduction in the amount of RA following engagement in the program was found, though the result was not significant. When Scott^36^ analyzed the difference in posttest scores in the treatment and control groups, a significant result was found. This indicates that the program did aid in reducing relationally aggressive behaviors. It is important to note that results from repeated measures analysis of variance found that participants did not believe that their relationally aggressive behaviors were diminished from engaging in the program. Therefore, caution is warranted when considering the use of the GWG intervention to reduce RA. This notion is further validated when examining results from the study by Stoll-Juredine^39^ on the effectiveness of GWG, as the findings suggest that the program was not successful in reducing relationally aggressive behavior. In this study, no statistically significant differences were found between pretest and posttest scores on student self-report of their engagement in RA (CSBS-S), their views of their peers’ engagement in RA (PBI), and teacher reports of the student participants’ engagement in RA (CSBS-T).^39^

Although the study by Stoll-Juredine^39^ did not yield significant results, Splett *et al.*,^38^ who similarly used the CSBS-S measure with the addition of the CSBS-T to analyze the GIRLSS intervention, did find significant reductions in relationally aggressive behavior. In this study, the CSBS-S and CSBS-T were used for self-report, teacher report, school counselor report, and averaged teacher and school counselor report, with the 2 latter reports yielding significant changes from pretest to posttest when comparing the participants in the treatment condition with participants in the control condition (95% CI [−1.8, −0.05] and [0.04, 1.31], respectively). Therefore, participants who engaged in the GIRLSS intervention demonstrated a greater change in relationally aggressive behavior per the school counselor and the averaged teacher and school counselor scores.^38^ However, the authors noted that caution should be taken when interpreting findings based on school counselor reports due to their investment in the success of the intervention.

A Friend In Deed is the final intervention based in an educational setting. Most participants (71%) found the lessons to be helpful. Furthermore, participants (89%) indicated that these lessons could potentially exert an influence on modifying their own relationally aggressive behavior.^35^ Although some participants were unavailable, an 8-month follow-up was conducted where the remaining participants responded to an additional questionnaire. The majority of participants who responded (74%) felt that the lessons heightened their awareness of both relational bullying and their own relationally aggressive behavior. Additionally, most of the respondents (75%) reported that the lessons had an impact on their behavior, such as how they chose to interact with their peers. Notably, participants were found to have a greater awareness of how their behavior impacted others at the 8-month follow-up compared with findings from the initial questionnaire.^35^

Sieving *et al.*^37^ made use of a variety of outcome measures, but regarding RA perpetration and victimization, 2 scales each including 6 items were adapted from the Multisite Violence Prevention Project.^65^ Participants who engaged in the Prime Time treatment condition were found to instigate significantly fewer relationally aggressive behaviors in the 30 days following treatment compared with participants in the control condition, yielding a small effect size. The study by Sieving *et al.*^37^ also provided qualitative evidence of reductions in relationally aggressive behavior, as case managers presented descriptions of participants’ experiences with the Prime Time intervention based on their self-reports. It was concluded from such descriptions that many participants had self-report responses that indicated linear decreases in relationally aggressive behaviors over time.^37^ The authors note how the focus of Prime Time on supportive relationships may have promoted reductions in relationally aggressive behavior.

In a pilot study conducted by Hoffman *et al.*,^34^ the effectiveness of the GRG intervention was evaluated using a sample of female adolescents housed in residential correctional facilities who had a reported history of aggressive behavior. Hoffman *et al.*^34^ assessed relationally aggressive behavior using the Relational and Direct Aggression Scale (RDAS).^60^ The RDAS was developed to evaluate RA and direct aggression by providing situations that typically occur in the lives of female adolescents.^34^^,^^60^ However, after participants engaged in the GRG intervention, there were no significant changes in levels of RA.

## Discussion

This review explored current interventions specifically designed for at-risk and incarcerated female youth with a focus on the effectiveness of said interventions. Findings may aid in understanding the elements necessary for providing successful intervention strategies for female juvenile offenders and at-risk female youth.

Studies reviewed in stages 1 and 2 used intervention strategies that stemmed from various theoretical models. As RA is more commonly exhibited by female adolescents, and the interventions discussed are aimed to reduce relationally aggressive behavior, it is imperative that the basis of such interventions acknowledge gender. RA can be a response to relationship dynamics, particularly for female adolescents whose well-being is closely linked to their connections with others, such that disruptions to their friendship networks can evoke a sense of dismay.^35^ A notable feature of the GRG intervention is its theoretical basis related to gender-specific socialization processes. The framework of the AMFJO intervention was constructed based on a preexisting anger program, with modifications to include gender sensitivity so that it was appropriate to use with female delinquents. Although the GRG and AMFJO interventions did not result in significant changes (potentially due to low power), their foundation and consideration of gender-specific processes are essential when aiming to treat RA as it is a behavior that manifests in distinct ways for female adolescents. RA interventions should continue to consider gender within their conceptual framework to ensure that strategies effectively address the nuanced ways in which social dynamics, power structures, and communication patterns manifest differently for female adolescents.

Both the JJAM and GIRLSS interventions used SIP models as a theoretical basis, which has significant merit as it can provide a comprehensive examination of the cognitive steps individuals undertake in social situations. The SIP model delves into the intricacies of how people encode, interpret, and respond to social cues, which sheds light on the nuanced cognitive processes that contribute to RA.^40^ By incorporating these insights into their interventions, a tailored approach can be taken to address the specific cognitive biases, attributions, and decision-making patterns associated with RA. As evidenced by the significant results of the JJAM and GIRLSS interventions, the use of the SIP model not only enhanced the quality of such interventions by providing a theoretical basis, but also fostered a deeper understanding of the cognitive deficits (eg, encoding, attributions, solution generation, and decision making) associated with relationally aggressive behaviors. More specifically, this is demonstrated by the positive outcomes associated with the hostile attribution bias mechanism of action in the JJAM study that relates to the SIP model. This bias may explain why female adolescents react in a relationally aggressive manner when perceiving an action as a threat to their sense of self. Therefore, using the SIP model allowed for more effective outcomes in mitigating RA within interpersonal relationships.

Many of the evaluated interventions exhibited similar key components. JAAM, which demonstrated significant efficacy, along with other reviewed interventions such as AMFJO, GRG, and A Friend In Deed, integrated psychoeducation into their programs. Similar to many established interventions, psychoeducation is recognized as a fundamental component of the treatment process, as it equips individuals with knowledge about their behaviors, fostering comprehension, self-awareness, and active engagement in the therapeutic journey.^66^

Two additional interventions, Prime Time and GWG, which demonstrated reductions in RA, incorporated the cultivation of social skills within their programs. This is an essential component as some experts suggest that the increased occurrence of aggressive responses during early and middle adolescence could stem from certain teenagers lacking the essential social skills to navigate interpersonal situations, leading them to resort to aggressive behavior.^67^^,^^68^ Consequently, the acquisition or reacquisition of these skills is crucial for their success in social settings. Additionally, the JJAM intervention, which yielded significant outcomes, emphasized the importance of problem solving. This emphasis on problem solving was also evident in the AMFJO and A Friend In Deed programs. Effective problem solving is crucial for adolescents, as those exhibiting RA may encounter challenges in understanding how to address issues appropriately. Finally, both AMFJO and GRG incorporated the use of coping skills into their interventions. Although neither AMFJO nor GRG yielded significant results, coping remains an integral component of numerous psychological treatments, particularly in the context of anger management.^69^

The inclusion of caregivers in interventions may allow for a more comprehensive approach that can aid in strengthening adherence and treatment effects. Notably, the GIRLSS treatment program, which yielded significant results, emphasized the crucial involvement of caregivers. This emphasis on family participation is particularly vital when working with adolescents, given the well-established role of family engagement as a mediating factor in treatment success.^70^ The authors of the GIRLSS study noted how the inclusion of caregivers allowed for the ability to modify disciplinary practices for relationally aggressive behaviors, thus contributing to the success of the intervention.^38^

On another note, incorporating elements of reward-based interventions, such as a point system, is known to enhance motivation for skill practice acquired during treatment, while also reinforcing the strengths and positive behaviors demonstrated by participants.^40^ The JJAM intervention, which yielded significant outcomes, used a point system for reinforcement of good behavior. The specifics of the point system of JJAM varied by facility but included extra phone time and cosmetic items as incentives for good behavior.^40^ Interestingly, the JJAM intervention not only incorporated an individual reinforcement system, but also implemented a group system. This approach not only encouraged members to practice the skills they learned, but also helped fulfill group expectations. By fostering positive peer pressure instead of negative interactions, this element of the JJAM intervention stood out.^40^

It is crucial to highlight that the GRG, JJAM, and GIRLSS interventions operate as manualized treatment approaches, setting the stage for prospective comparative studies. Notably, both JJAM and GRG interventions underwent evaluation with a focus on female adolescents within correctional facilities and therefore were the most relevant to the initial impetus for this review. Despite this alignment, the JJAM study produced significant results, whereas the GRG study did not yield such outcomes. In contrast, the GIRLSS intervention, implemented within a school setting, demonstrated success in curbing relationally aggressive behavior. Consequently, the GIRLSS intervention emerges as a promising and effective manualized treatment approach for mitigating RA among at-risk female youth.

The settings in which interventions were conducted may have influenced their effectiveness. Individuals in correctional facilities may exhibit more severe forms of relationally aggressive behavior, posing challenges for achieving positive changes in RA. This difficulty is reflected in the lack of significant findings observed in 2 of the 3 interventions implemented in a juvenile justice facility. On the other hand, interventions in a school setting may encounter less severe forms of RA, potentially facilitating changes. However, the school environment itself presents challenges, as participants may not be fully focused on interventions conducted throughout the school day, which is evident by findings of 3 of the 4 school-based interventions failing to yield desired outcomes. Notably, GIRLSS, a school-based intervention, achieved significant results, possibly attributable to its longer session duration of 1.5 hours over an extended period. Remarkably, the one clinic-based intervention, Prime Time, achieved significant results, likely owing to willingness of participants to engage in treatment, as they actively tried to attend. Nevertheless, the extended duration of this intervention, spanning 18 months, could have impacted the ability of the Prime Time intervention to achieve desired outcomes.

It is important to note that studies in stages 1 and 2 employed diverse outcome measures. As previously mentioned, the variation in the conceptualization of RA can lead to the use of a range of measurement techniques to assess the construct, which affects the determination of the effectiveness of interventions reviewed. The GWG study and GIRLSS intervention stood out for including individuals closely connected to the participants in their assessment (ie, parents and teachers), which provided an objective perspective and reduced the reliance on self-reports. In the GRG study, 4 measures were employed, and each had subscales that addressed different forms of aggression, with one measure used to directly assess RA and direct aggression.^34^ Notably, 6 measures were used to assess the success of the JJAM intervention and allowed for conclusions to be drawn on different forms of RA (general, proactive, reactive, and indirect).^40^ Therefore, the measures used to assess the JJAM intervention appear to be particularly effective in conveying relevant results.

As previously highlighted, a considerable number of current aggression treatments were initially formulated for male adolescents, potentially resulting in an insufficient focus on addressing the distinct needs of female adolescents. Therefore, in formulating interventions for RA, it is essential to incorporate a gender-sensitive theoretical framework to ensure that strategies are tailored to effectively address factors that are unique to female participants. Moreover, based on findings from this review, it appears that employing SIP as a theoretical foundation is the most impactful approach for interventions targeting RA. Given that RA frequently occurs in social contexts where social dynamics are crucial, incorporating elements of the SIP model can assist in understanding and responding to social cues more effectively.

Considering the setting in which RA occurs, coupled with the observation that youth employing RA frequently exhibit deficiencies in peer interaction skills, the integration of social skills training becomes a crucial element in interventions. Social skills training could empower youth who display relationally aggressive behaviors to develop effective communication and empathy, thereby promoting positive social interactions. Additionally, incorporating problem solving as a component in RA interventions may provide adolescents with essential skills to navigate social conflicts, such as generating adaptive solutions to social problems and building adaptive goal-attainment skills.^24^

While these components are effective on their own in treating RA, for sustained progress, it is vital for treatment programs to integrate supplementary elements that reinforce the application of learned techniques. A component presented in certain interventions, which possibly contributed to the efficacy of the treatment, was the implementation of a reward system. This not only potentially enhanced the immediate effectiveness, but also may have played a pivotal role in maintaining and sustaining the skills learned over time. The incorporation of a group reinforcement system seen in the JJAM intervention is noteworthy given that RA interventions often involve addressing negative peer interactions. This specific element could have played a key role in the success of JJAM and thus had the potential to contribute to the promotion of positive dynamics.

Additionally, as the emphasis of this review was on evaluating interventions for female juvenile offenders and at-risk female adolescents, it is important to recognize the potential challenges in treating individuals who make up these populations. For example, female juvenile offenders commonly exhibit low motivation for change, trust issues, noncompliance, and elevated levels of anger and impulsivity, all of which have been linked to suboptimal treatment responses.^71^, ^72^, ^73^ Addressing the unique barriers and obstacles associated with these groups is essential when developing interventions tailored to their needs.

This 2-stage review of intervention strategies designed to treat RA has limitations. Much of the research addressing the treatment of RA lacks the necessary specificity when it comes to the juvenile justice population, which in turn has contributed to a limited yield of relevant results. The outcomes obtained during the initial stage of this review often revolved around repeated analyses of the same intervention. Thus, the scope was extended to include individuals considered at risk, aiming to enhance the breadth of available information, although some limitations on the available data persisted. Moreover, among the complete set of articles gathered from our initial searches, we discovered that most of the research pertaining to RA focused primarily on the behavior itself, rather than delving into the development or effective implementation of interventions aimed at addressing this topic or treating associated behaviors. Finally, it is important to acknowledge that the literature highlights variations in the manifestation of relationally aggressive behaviors based on an individual’s developmental age.^4^ Consequently, a limitation of this study is the exclusion of participants younger than 10 years of age, as we potentially excluded an extensive body of literature^74^ focused on elementary and middle school–age children who display RA. Expanding the acceptable age range criteria for this review might have offered a more comprehensive examination of interventions designed to treat RA.

Despite the limitations in the field of RA research, the information gathered from this review provides several promising directions for future studies. First and foremost, some articles included in this review noted concern over the ability of their chosen treatment to effectively translate across diverse groups of female adolescents. This uncertainty offers an interesting opportunity for future studies. By investigating how treatments transition across different contexts, researchers may better understand how interventions can be adjusted to suit the specific needs of diverse subsets of female adolescents displaying RA. Finally, there is a notable and pressing demand for the development and validation of standardized measures that focus explicitly on RA. Many of the articles reviewed used various instruments that included only subscales to measure RA or measured the construct in conjunction with another psychologically related behavior. Before inclusion in a study assessing the efficacy of interventions, newly developed measures targeting RA should undergo a rigorous testing process.

Moreover, future research should strive to enroll samples of sufficient size to yield statistically significant results if they exist, as many of the studies reviewed were hindered by low power. This approach would enhance the ability to assess the effectiveness of RA interventions more efficaciously. Overall, there are promising advancements in the development and implementation of interventions tailored for treating relationally aggressive behaviors seen in female youth deemed at risk or currently in the juvenile justice system.

## CRediT authorship contribution statement

**Jenny Magram:** Data curation, Conceptualization. **Erica Ackerman:** Data curation, Conceptualization. **Claire Stafford:** Resources. **Tom D. Kennedy:** Supervision, Conceptualization.
